# The Spitting Animal

**Published:** 1897-06

**Authors:** 


### The Spitting Animal.

For many years past travelers from Europe have been accustomed to inveigh against the American people for the extreme prevalence of that most disgusting habit, the continual spitting in public places. Many writers at home have joined in the outcry. Yet no headway was made. The evil seems incurable. Even ten years ago the most ardent reformers were hopeless; nevertheless within the current decade, active steps have been taken, and wonderful progress made in stamping out the filthy habit.

The knowledge that consumption is a preventable disease is the root cause of this noted social reform. All the people dread what has been well styled "the white plague." All are deeply concerned in its abatement. Hence the announcement by the medical profession that "spittle" is by far the chief factor in the spread of consumption, has called universal attention to the enormous magnitude of the evil habit. Multitudes deaf to considerations of decency become wide awake when life is at stake.

So it has come to pass that newspapers and popular magazines are constantly co-operating with medical and sanitary periodicals in fighting the detestable practice. Thus public men have been forced into line. In quite a number of the Eastern cities laws have been passed which are educating the people and limiting the evil. On street cars and ferry boats are seen placards rigidly forbidding spitting, The regulation is strictly enforced, popular opinion sustaining it. Some cities have passed ordinances forbidding spitting upon sidewalks. The movement goes on. It is high time that our cities should fall in line, and our street cars and sidewalks be set free from a dangerous cause of disease, which is also an offense to common decency.—*Ex.*

A NUMBER of Baltimore physicians, dentists, and druggists have taken steps to either lessen the number of dispensaries, or to compel them to limit themselves in the number of cases they treat, and to those to whom they give free treatment.

